# Intraoperative Tumor Detection Using Pafolacianine

**DOI:** 10.3390/ijms232112842

**Published:** 2022-10-25

**Authors:** Mihaela Elisabeta Dindere, Antoanela Tanca, Mihaela Rusu, Elisa Anamaria Liehn, Octavian Bucur

**Affiliations:** 1 Victor Babes National Institute of Pathology, 050096 Bucharest, Romania; 2 Faculty of Medicine, Carol Davila University of Medicine and Pharmacy, 050474 Bucharest, Romania; 3 Department for Cardiology, Angiology and Internal Intensive Care, Medical Faculty, RWTH Aachen University, 5207 Aachen, Germany; 4 Institute for Molecular Medicine, University of Southern Denmark, 5230 Odense, Denmark; 5 Viron Molecular Medicine Institute, Boston, MA 02108, USA

**Keywords:** pafolacianine, intraoperative, tumor detection, folate receptor, fluorescent imaging, near-infrared spectrum

## Abstract

Cancer is a leading cause of death worldwide, with increasing numbers of new cases each year. For the vast majority of cancer patients, surgery is the most effective procedure for the complete removal of the malignant tissue. However, relapse due to the incomplete resection of the tumor occurs very often, as the surgeon must rely primarily on visual and tactile feedback. Intraoperative near-infrared imaging with pafolacianine is a newly developed technology designed for cancer detection during surgery, which has been proven to show excellent results in terms of safety and efficacy. Therefore, pafolacianine was approved by the U.S. Food and Drug Administration (FDA) on 29 November 2021, as an additional approach that can be used to identify malignant lesions and to ensure the total resection of the tumors in ovarian cancer patients. Currently, various studies have demonstrated the positive effects of pafolacianine’s use in a wide variety of other malignancies, with promising results expected in further research. This review focuses on the applications of the FDA-approved pafolacianine for the accurate intraoperative detection of malignant tissues. The cancer-targeting fluorescent ligands can shift the paradigm of surgical oncology by enabling the visualization of cancer lesions that are difficult to detect by inspection or palpation. The enhanced detection and removal of hard-to-detect cancer tissues during surgery will lead to remarkable outcomes for cancer patients and society, specifically by decreasing the cancer relapse rate, increasing the life expectancy and quality of life, and decreasing future rates of hospitalization, interventions, and costs.

## 1. Introduction

Cancer is one of the major causes of death worldwide. According to recent statistics, there were ~10 million deaths in 2020 [1] and ~9.6 million deaths in 2018 [2] due to cancer, with ~18 million new cases diagnosed in 2020 [1] and ~18.1 million new cases in 2018 [2]. The number of cancer-related deaths is expected to increase to 16.4 million by 2040, with ~30 million new cases/year [2].

Surgery is the most effective procedure for completely removing localized cancer, and it may also be used to treat metastasized malignancies [3]. Despite the progress in preoperative imaging techniques (X-rays, positron emission tomography (PET), computed tomography (CT) scans, magnetic resonance imaging (MRI), ultrasound, etc.), during surgery, the oncological surgeon must rely especially on visual inspection and palpation to identify the cancerous lesions [4]. 

As the surgical field moves towards minimally invasive surgery and robotic-assisted surgery, the loss of palpation requires a supplementary cancer detection method, making the use of intraoperative imaging techniques (such as those based on the use of fluorescent agents) increasingly important [5]. 

In this scenario, with the knowledge that the most frequent cause of the recurrence of many malignancies is the incomplete excision of the tumor, an imaging system that allows the surgeon to identify the cancerous tissue in real time, without compromising the surgical field, could produce a significant shift in surgical oncology [4,5]. 

An imaging system of this kind can be provided by the fluorescent agents, such as pafolacianine, also known as OTL38. Pafolacianine was first developed by Philip S. Low, PhD and Presidential Scholar for Drug Discovery, and Ralph C. Corley, Distinguished Professor of Chemistry at Purdue University, the pioneer of this project, in 2001, when excellent results were obtained in mice. Even though pafolacianine had promising potential, the transition to human trials was met with skepticism by surgeons who considered the methods for detecting cancer to be accurate enough [5]. 

Therefore, after years of intense research on this topic and successful clinical trials, pafolacianine (Cytalux, On Target Laboratories, LLC), a fluorescent imaging agent for adult patients with ovarian cancer, was approved by the U.S. Food and Drug Administration (FDA) on 29 November 2021 as an additional approach to improved intraoperative tumor detection, being used with a near-infrared fluorescence imaging system specified by the FDA as suitable for use with pafolacianine [6]. 

Pafolacianine is a folate analogue conjugated with an indocyanine-green-related fluorescent dye called SO456, which absorbs light in the near-infrared spectrum [7,8]. Light absorption occurs within a range from 760 nm to 785 nm, with a maximal absorption of 776 nm, while the fluorescent emission occurs within a range from 790 nm to 805nm, with a maximal emission of 796 nm [7]. Intraoperatively, the fluorescent dye within the agent lights up when a special camera system (a near-infrared imaging system) is used in fluorescent-guided surgery. Near-infrared imaging is a cutting-edge technology using a dedicated camera system that can detect fluorescence in the millisecond range, without interfering with the surgeons’ ability to perform properly [4]. Near-infrared fluorescence has the advantage of a deeper tissue penetration and low autofluorescence compared with shorter wavelengths, which enables the advanced imaging and detection of solid tumors [9]. 

**Figure 1:**
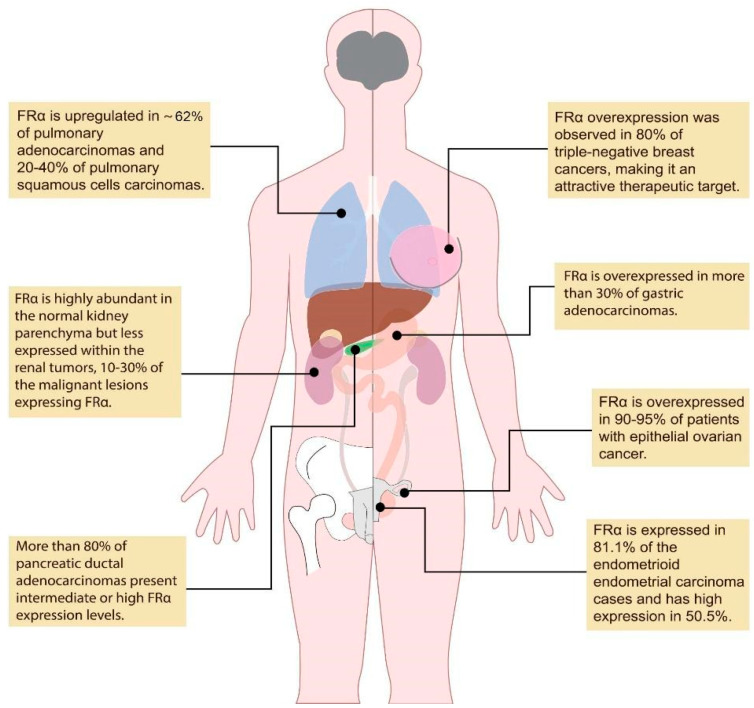
Overexpression of FRα in different types of malignancies. (created based on the information from references [11,12,13,14,15,16,17,18,19,20,21,22]).

Pafolacianine targets the folate receptor alpha (FRα), a membrane glycoprotein anchored by glycosylphosphatidylinositol molecules that is usually found in a certain cluster of polarized epithelial cells within healthy tissues, such as the kidney and choroid plexus [10]. The FRα is also overexpressed in a wide variety of malignancies, with some studies citing about 40% of malignant lesions [5], such as ovarian cancer, invasive pulmonary adenocarcinoma, and adenocarcinoma spectrum lesions in the lung (Figure 1) [11,12,13,14,15,16,17,18,19,20,21,22]. 

The distribution pattern of FRα and the excellent tumor contrast obtained using pafolacianine in both murine cancer models and human malignancies make this combination an attractive target for diagnostic and therapeutic development [13,23]. Thus, it was approved by the FDA for use in clinical practice during surgery [6]. 

This review emphasizes the applications of the FDA-approved pafolacianine for the precise intraoperative detection of cancer lesions. As evidenced by previous published reviews, the near-infrared fluorescent agents, such as pafolacianine, as a supplementary approach in surgical oncology, can provide a promising alternative to the conventional intraoperative methods, such as inspection and palpation [5,9,10,16]. The enhanced visualization and removal of hard-to-detect cancer tissues during surgery will lead to remarkable outcomes for cancer patients and society by decreasing the cancer relapse rate, increasing the life expectancy and quality of life, and decreasing rates of hospitalization, interventions, and costs.

## 2. FDA-Approved Pafolacianine for Intraoperative Ovarian Cancer Detection

Epithelial ovarian cancer (EOC), also known as the ‘silent lady killer’ [24], ranks fifth in cancer deaths among women, and it is the main cause of death of all gynecological malignancies in both Europe [25] and the United States [26]. Globally, the 5-year survival rate is 45% [27] and only 20–25% for the late stages of ovarian cancer [28,29]. 

The unclear and nonspecific clinical signs in the early stages of ovarian cancer, combined with the absence of a reliable screening tool, often leads to an advanced-stage diagnosis [24]. Currently, the most effective treatment for advanced-stage ovarian malignancies (i.e., International Federation of Gynecology and Obstetrics stage IIb to stage IV) usually consists of cytoreductive surgery followed by combination chemotherapy [4]. Several studies have demonstrated that the degree of cytoreduction and the amount of residual tumor deposits that remain following cytoreductive surgery are the most meaningful prognostic indicators of survival and some of the few prognostic factors that can be directly influenced by the surgeon [30,31,32,33,34]. 

In contrast with the preoperative radiologic approaches, which are not tumor-specific, fluorescence imaging techniques offer a higher resolution and sensitivity [24]. These methods can provide real-time feedback that may lead to an increased number of resected metastatic lesions and more thorough resection [4]. Thus, intraoperative tumor-targeting imaging can enable the better visualization and removal of hard-to-detect lesions, having a real impact on the entire surgical oncology field. 

The overexpression of FRα in 90–95% of patients with EOC [14,15] and the absence of FRα on normal cells, reflecting a high tumor-to-background ratio (TBR), offer an excellent opportunity to apply near-infrared imaging in patients with ovarian cancer for the purpose of upgraded intraoperative tumor detection and the radical excision of the malignant tissue, thereby improving ovarian cancer outcomes [24]. This technology allows for an engineered approach in terms of bettering the cancer staging process and the technique of cytoreduction, additionally leading to effective debulking surgery in the hyperthermic intraperitoneal chemotherapy procedure [35] for solid tumors with a peritoneal dissemination pattern [36,37] and future patient-tailored surgical interventions. 

According to a phase I clinical trial conducted based on 30 healthy volunteers, pafolacianine caused mild and easily manageable hypersensitivity. These reactions were probably related to the aggregation of pafolacianine rather than reflecting a typical allergic response to the substance, suggesting that the severity may be diminished by adjusting the dose and the dilution of the drug. When translated to 12 patients with ovarian cancer, pafolacianine enabled surgeons to detect malignant lesions with high sensitivity and specificity, with the surgeons being able to detect 29% of all resected cancer lesions that could have been missed without the use of near-infrared fluorescence imaging [4]. The study also reported the mild homogeneous fluorescence of the uterus and fallopian tubes, which moderately express FRα, but this was clearly distinct from the fluorescence of the malignant ovarian tissue [38,39]. In most patients, the lymph nodes were brightly fluorescent, but only a few contained ovarian cancer metastases due to the binding of pafolacianine to the folate receptor β (FRβ), expressed on the surface of the activated macrophages within the non-cancerous lymph nodes [40,41,42,43], representing 56% of all false positive lesions [4]. This apparent drawback of false positive fluorescence can, in fact, be helpful, because activated macrophages can be tumor-associated macrophages, which play a role in tumor expansion [44,45,46]. 

The study also reported other advantages of fluorescence imaging with pafolacianine, including 1 cm deep tissue penetration, a low autofluorescence of normal tissues when excited by the near-infrared light, a long tumor residence, and fast plasma clearance [4].

The research on near-infrared imaging using pafolacianine in ovarian cancer patients continued with a phase II clinical study (NCT02317705, see Table 1), which enrolled 29 women over the age of 18 with known or suspected ovarian cancer, scheduled for cytoreductive surgery. The results showed that, when assuming a possible correlation of detection between multiple lesions within the patient, pafolacianine had a 97.97% sensitivity (95% lower boundary CI = 87.75) and 94.93% positive predictive value (lower boundary CI = 86.16). Additional analyses revealed that 48.3% of the patients (95% CI, 0.29–0.67) had at least one malignant lesion detected by fluorescent imaging with pafolacianine that would have been undetected by the surgeon. The toxicity was similar to that observed in the preceding phase I study, with all patients having at least one adverse reaction, such as procedural pain, vomiting, abdominal pain, or nausea [47]. Additionally, most of the false positive lesions were located in the lymph nodes due to the FRβ expression in the macrophages [48,49].

Later on, data collected for the FDA approval were used to evaluate the safety and efficacy of pafolacianine in a phase III clinical trial (NCT03180307; see Table 1), which included 178 women with clinical suspicion or a diagnosis of ovarian cancer. The patients were scheduled to undergo cytoreductive surgery, with interval debulking surgery for recurrent ovarian tumors. All of them received pafolacianine, and only 134 (aged 33 to 81) received intraoperative fluorescence imaging as an additional method to the standard preoperative imaging techniques and the intraoperative visualization and palpation in visible light. The study highlighted that, among these patients, 36 (26.9%) had at least one malignant lesion observed with pafolacianine that was not detected visually or by touch. The false positive rate of the near-infrared fluorescent imaging with pafolacianine was 20.2% (95 %CI 13.7, 28.0%) compared to the accurate detection of cancer tissue confirmed by central pathology. 

In terms of safety, the toxicity was minor, and the most prevalent adverse effects (≥1%) in patients included vomiting, dyspepsia, nausea, chest discomfort, abdominal pain, flushing, pruritus, and hypersensitivity. Pafolacianine may also cause fetal harm in the case of its administration to pregnant women. 

**Figure 2:**
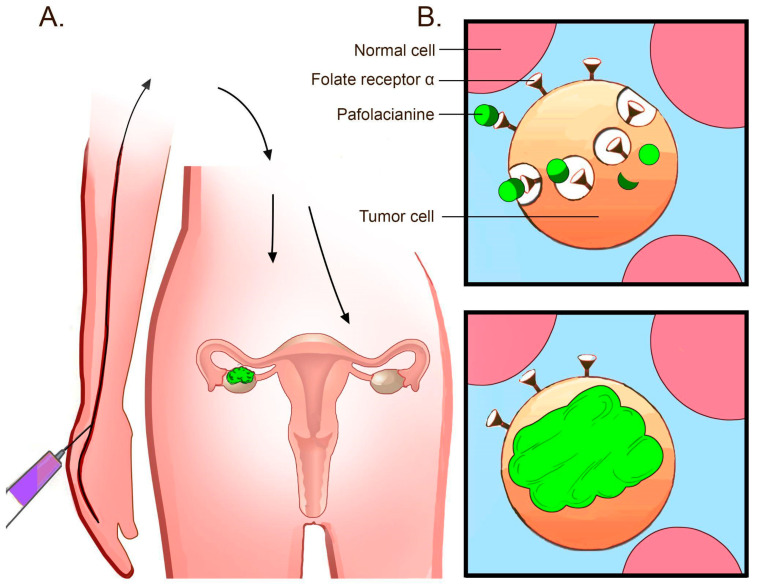
Mechanism of the pafolacianine-based intraoperative detection of tumors. (**A**) Pafolacianine is intravenously administered within a couple of hours before the surgery. As the agent circulates through the blood flow, only the tissues that overexpress FRα capture the dye. FRα upregulation in ovarian cancer lesions enables the binding of pafolacianine and near-infrared fluorescence imaging during surgery. (**B**) Pafolacianine binds FRα overexpressed in ovarian cancer tumors. The dye accumulates in the cells via endocytosis [6].

According to the FDA, pafolacianine is intravenously administered (see Figure 2) over 60 min at the recommended dose of 0.025 mg/kg 1 to 9 h prior to the surgery, avoiding the use of folate, folic acid, or folate-based medications during the 48 h before the administration of pafolacianine [6]. 

As the agent circulates through the bloodstream, only the tissues strongly expressing FRα capture the dye, while the non-expressing tissues clear the ligand very quickly within a couple of hours. The amount retained in the malignant lesions then guides the surgeon to ensure the precise excision of the tumor [5]. 

These encouraging results, together with the FDA approval of pafolacianine for intraoperative fluorescent imaging, will pave the way to the improved staging of ovarian cancer, the complete removal of the malignant tissues during surgery, and enhanced outcomes in ovarian cancer patients.

## 3. Pafolacianine for the Detection of a Wide Variety of Malignancies during Surgery

There is a wide range of cancers that overexpress FRα, such as lung cancer, triple-negative breast cancer, gastric cancer, and endometrial cancer. It seems that approximately 40% of human malignancies overexpress FRα, making fluorescent imaging with pafolacianine tremendously useful [5] (Table 1).

Since folate receptor-α is usually expressed both in primary tumors and in metastases, pafolacianine could also be used to surgically remove metastatic cancer lesions in a variety of malignancies [11,12,13,22].

### 3.1. Lung Cancer

Lung cancer is the most frequent and lethal type of cancer globally. The vast majority (85%) of cases of all lung cancers are represented by non-small cell lung cancer (NSCLC), the cancer with the highest mortality in the USA [50]. 

Despite attempts to surgically remove the malignant lesions, almost 40% of lung cancer patients succumb to disease relapse within 5 years of oncologic excision [51]. Recent data report that 10–20% of NSCLC patients develop synchronous disease that is routinely undetected during surgery due to the limitations of both visual and tactile feedback [51].

Currently, there are many technologies aiming to improve the conventional intraoperative visualization and palpation methods in order to identify pulmonary nodules and ground-glass opacities (GGOs, hazy radiographic findings representing inflammatory processes, benign lesions, or specific types of lung malignancies [52]), including intraoperative ultrasound, radionucleotide imaging, intraoperative marking by bronchoscopy, wire localization, and high-resolution computed tomography [13].

Unfortunately, these techniques have considerable drawbacks and limitations. They require presumptive information about the nodule’s localization, harbor morbidity, and risk of complications (hemothorax, pneumothorax, or air embolus) and fail to identify synchronous disease or to accurately assess the margin status in real time [13] because of GGOs’ subtle changes in the parenchymal architecture [53]. 

As modern surgery advances towards the use of safe and effective minimally invasive operative approaches, the development of high-specificity molecular contrast agents and real-time fluorescent imaging during surgery will represent a promising alternative strategy that can be used to localize small nodules, to identify primary metastases, and to improve margin assessment [54,55]. 

The presence of FRα upregulation in 62% of pulmonary adenocarcinomas makes intraoperative molecular imaging with pafolacianine an important tool for identifying adenocarcinoma spectrum lesions, additional sub-centimeter neoplastic processes, occult tumors, and small nodules [13]. It has been also demonstrated that 20–40% of pulmonary squamous cell carcinomas express FRα, highlighting pafolacianine as an excellent candidate for intraoperative fluorescent imaging in most pulmonary adenocarcinoma patients and almost one-third of those patients with other types of NSCLC [11,12].

Studies report that, for oncological resection in lung cancer patients, the FRα expression is independent of several factors, such as age, gender, race, smoking history, or the cancer stage [13], and what is more, these variables do not have any statistical relevance in predicting TBR [56]. However, there seems to be a negative correlation between in situ TBR measurement and the depth of the lesion in the lung tissue [56]. 

To date, near-infrared imaging with pafolacianine has shown excellent feasibility in lung cancer patients, facilitating tumor localization and a similar capacity for margin estimation to pathologic evaluation, yielding real-time data that is useful for patients with small or peripheral lesions [57]. 

According to Newton et al., intraoperative molecular imaging after pafolacianine injection was able to identify 15 out of 15 sub-centimeter malignant pulmonary nodules, all of them being fluorescent, while fluorodeoxyglucose-positron emission tomography (FDG-PET) only identified 26.7% of them [58].

Thus, pafolacianine has been proven to show adequate safety and efficacy in lung cancer patients, improving the localization of hard-to-detect lesions and enabling precise real-time margin evaluation before pathologic analysis. Intraoperative molecular imaging ultimately serves as a very useful method for detecting small pulmonary nodules. In addition to these advantages, no additional invasive procedures are needed, intraoperative interpretation is achieved rapidly (within 5 min), and the drug provides no toxicity [57]. 

### 3.2. Renal Cancer 

In contrast with the pattern presented in ovarian and lung cancer, FRα is highly abundant in the healthy kidney parenchyma (up to 100% expression on the apical surface of the proximal tubules, providing physiologic folate reabsorption) [16] but less expressed within renal tumors [17,38], which appear dark (10–30% staining), while the surrounding normal tissue is brightly fluorescent [18]. 

There are also studies that report the much higher expression of FRα in metastatic renal cell carcinoma (RCC) compared to small kidney tumors, making FRα upregulation a potential target for RRC prognostication [59].

In the case of the small renal tumors, the preferred procedure is partial nephrectomy, since it aims at preserving the function of the nephrons and kidney tissues as much as possible, while focusing on the removal of the target tumor tissue. During surgery, the surgeon must remove the malignant tissue, providing an adequate margin around the lesion without resecting too much of the normal parenchyma and limiting the generated ischemia [60]. 

With respect to these requirements, near-infrared imaging is the best option for the visual demarcation of the cancer tissue [61,62], especially with the help of robotic assistance, a common procedure used among urologists [63,64] which enables additional dexterity and three-dimensional laparoscopic visualization [65,66]. At present, several studies have shown encouraging results based on near-infrared imaging during partial nephrectomy in regard to the technique’s capacity for easy tumor identification due to the high fluorescence contrast and the assessment of the intact margins of healthy parenchyma surrounding the resected malignant lesions, as an indicator of the complete removal of the tumor [60]. 

### 3.3. Pituitary Tumors

Pituitary adenomas represent approximately 10% of all intracranial tumors [67] and present a unique challenge to the achievement of complete removal while preserving the function [68]. However, the relapse rate can rise up to 20% after surgery due to the incomplete excision of the tumor [67].

Therefore, the use of a developing technique such as fluorescent imaging during surgery could enable neurosurgeons to achieve a more precise visualization of the malignant lesions due to an excellent contrast with the healthy background [67]. 

Currently, several studies have highlighted the presence of FRα upregulation in a large majority of nonfunctional pituitary adenomas with strong fluorescence using pafolacianine, while the functional adenomas do not overexpress FRα and have a very low near-infrared fluorescence [69,70,71,72]. 

For the nonfunctional adenomas, pafolacianine demonstrated an approximately 75% sensitivity, 100% specificity, 100% positive predictive value, and 62% negative predictive value, and it estimated the margins with 100% accuracy. The sensitivity may also reach 100% in those nonfunctional adenomas which overexpress FRα [73,74,75]. 

Additionally, the absence of fluorescent tissue after near-infrared-guided surgery is strongly correlated with the complete resection of the pituitary adenoma based on the postoperative MRI findings, especially when surgery is performed using angled near-infrared endoscopes [67].

### 3.4. Gastric Cancer

Gastric cancer is the fifth most common neoplasm and the third most fatal cancer worldwide [76].

Usually, the staging of gastric adenocarcinoma is achieved using cross-sectional imaging, endoscopic ultrasound, and diagnostic laparoscopy with peritoneal washings [77]. According to the National Comprehensive Cancer Network guidelines, gastric resection is recommended for localized malignancies (T1-2N0), while the removal of locally spread cancer lesions should be preceded by neoadjuvant chemotherapy [78]. 

However, there are many limitations regarding these staging techniques and intraoperative approaches to gastric tumors, including the limitations on the performance of endoscopic ultrasound [79,80,81], diagnostic laparoscopy with peritoneal washings, surgical lymph node staging, the imaging evaluation of the neoadjuvant therapy response, and the intraoperative pathologic assessment of frozen sections [22].

These drawbacks and others can lead to the identification of new strategies and technologies that may perfect cancer diagnosis and staging [22,82,83,84,85,86,87,88,89], increase the level of tumor localization, and improve margin assessment during surgery [22].

Recent studies have highlighted the overexpression of FRα in over one-third of gastric adenocarcinomas [16], making near-infrared imaging with pafolacianine an attractive approach for the surgically treatment of gastric malignancies [22].

This method revealed several advantages when tested for the improvement of the surgical management of gastric tumors, such as easy administration; the renal excretion of the agent, which minimizes the background fluorescence observed when using hepatically excreted drugs; a good depth of penetration, allowing for the visualization of even T3 tumors, which do not penetrate the gastric wall; visible fluorescence even at low levels of FRα expression; and a lack of associated toxicity. The fluorescent dye also accumulates in the lymph nodes, but no macroscopic discrimination was observed between metastatic and benign lymph nodes [90,91]. 

Therefore, intraoperative molecular imaging with pafolacianine seems to have great potential to provide the improved staging and laparoscopic diagnosis of gastric cancer patients, the precise assessment of tumor expansion and regional lymphatic metastasis, as well as the better selection of patients for neoadjuvant chemotherapy [22].

There are also studies that have evaluated the feasibility of intraoperative FRα-targeted tumor detection with pafolacianine in other types of cancers, such as endometrial cancer and pulmonary osteosarcoma metastases [92,93]. 

Currently, auspicious results have been highlighted in a large variety of malignancies, but further research should confirm these findings and investigate intraoperative near-infrared imaging with pafolacianine in the remaining types of cancers that overexpress FRα. 

## 4. Pafolacianine in Comparison with Other Similar Agents Used for Intraoperative Molecular Imaging

Over time, a multitude of other substances have been tested that specifically bind cancer cells and provide a suitable contrast and depth of penetration in order to guide the surgeon in performing a complete resection of the tumor during surgery.

Generally, agents that emit fluorescence in the visible range (400–700 nm), such as 5-aminolevulinic acid, folate-fluorescein isothiocyanate, and sodium fluorescein, exhibit observable levels of background autofluorescence, provide poor tissue penetration, and have higher scattering [91] compared with the near-infrared ligands (700–850 nm), such as pafolacianine and indocyanine green, which are showing increasingly promising results in cancer targeting during surgery [67].

In terms of safety, the majority of agents used for intraoperative tumor detection nowadays have a low toxicity profile and exert few to no side effects [4,93,94] (see Table 2). 

In addition to these already FDA-approved molecular tracers used for fluorescent guided surgery, according to a recent review by Barth et al., a total of 39 contrast agents used for tumor-specific targeting are being studied in over 85 clinical trials in the US alone. Three of these novel probes (BLZ 100, LUM 015, and SGM-101) have reached phase III clinical trials and are expected to be approved by the FDA in a couple of years [95,96]. BLZ 100, used for real-time tumor detection during brain and breast cancer surgeries, consists of a natural chlorotoxin peptide, which targets the protein components of cholesterol-rich lipid rafts from cancer lesions [97]. LUM 015 contains a Cy5 fluorophore linked to a cathepsin activatable peptide, being used in combination with the LUM imaging system for fluorescent-guided surgery in residual breast cancer, gastrointestinal cancer, and prostate cancer [98]. SGM-101, an antibody–dye conjugate composed of a fluorochrome, BM104, linked to a chimeric monoclonal antibody binding the carcinoembryonic antigen, enables intraoperative fluorescent imaging in rectal and colorectal cancer patients [99].

Thus, over the past two decades, the multidisciplinary collaboration between biomedical researchers has nurtured the development of advanced imaging instrumentation, novel tumor-specific tracers, and targetable biomarkers in order to perfect fluorescent-guided surgery.

## 5. Benefits and Limitations of Using Pafolacianine for Cancer Detection during Surgery

Near-infrared imaging using pafolacianine for cancer detection during surgery has several benefits in terms of its safety and efficacy (see Table 3) that can provide significant changes in cancer patients’ outcomes, especially in light of the progress in oncological research and minimally invasive surgery. 

The benefits include easy administration shortly before the surgery, a low toxicity profile with mild adverse reactions, a high affinity, excellent contrast, excellent tumor visualization during surgery, good tissue penetration, and rapid plasma clearance.

Regarding the limitations, intraoperative imaging with pafolacianine, as a newly developed technology, has minor downsides, such as the need to become more popular among both surgeons and patients, the risk of fetal harm, the avoidance of folate-based drugs, and the occurrence of false positive and false negative results. However, these limitations are combined with the method’s great potential to be perfected in further studies [6].

## 6. Conclusions

Since the surgical removal of malignant lesions is the most adequate curative option for many cancer patients, new methods that provide the surgeon with the capacity for the accurate detection of the tumors during surgery are extremely useful.

With the evolution of minimally invasive surgery and robotic-assisted surgery, intraoperative imaging procedures have attracted great interest in the field of oncological research. Thus, the FDA’s approval of pafolacianine, a near-infrared imaging agent with excellent safety and efficacy properties, represents a step closer to the complete resection of tumors and, ultimately, to a decreased recurrence rate and improved outcomes in ovarian cancer patients. To date, multiple studies have highlighted the beneficial use of pafolacianine in several types of malignancies that overexpress FRα. However, future research must confirm these optimistic results, and more comprehensive studies are required.

## Figures and Tables

**Table 1:** Clinical trials with pafolacianine for intraoperative cancer detection.

ClinicalTrials.gov	Study Title	Condition	Status	Locations
Ovarian cancer detection				
NCT02317705	Phase 2 Study of OTL38 for Intra-operative Imaging of Folate Receptor-alpha Positive Ovarian Cancer	Ovarian cancer	Completed	University of CA at Irvine, CA, USA; Moffitt Cancer Center Tampa, FL USA; Mayo Clinic, Rochester, MI, USA; University of Pennsylvania, PA, USA
NCT03180307	OTL38 for Intra-Operative Imaging of Folate Receptor Positive Ovarian Cancer	Ovarian cancer	Completed	The Mayo Clinic, Phoenix, AZ, USA; University of Arizona, Tucson, AZ, USA; City of Hope Medical Center; Duarte, CA, USA (and 8 others)
NCT04941378	OTL38 Injection (OTL38) for Intra-Operative Imaging of Folate Receptor Positive Ovarian Cancer	Ovarian cancer	Withdrawn	Abramson Cancer Center, University of Pennsylvania, Philadelphia, PA, USA
Other cancers/diseases				
NCT02602119	Intraoperative Imaging of Pulmonary Nodules by OTL38	Neoplasms	Completed	Hospital of the University of Pennsylvania, Philadelphia, PA, USA
NCT03938701	Fluorescence Imaging of Disease Activity in IBD and Rheumatoid Arthritis Using OTL38	Inflammatory bowel disease, Rheumatoid arthritis	Not yet recruiting	University Medical Center Groningen, Netherlands
NCT02872701	OTL38 Injection for Intraoperative Imaging of Folate Receptor Positive Lung Nodules	Lung neoplasms	Completed	Beth Israel Deaconess Medical Center Boston, MA, USA; Cleveland Clinic Cleveland, OH, USA; University of Pennsylvania, Philadelphia, PA, USA (and 3 others)
NCT02852252	Solid Tumor Cancer Surgery with or without Intraoperative Imaging: A Registry	Bladder cancer, Gastric cancer	Completed	Hospital of the University of Pennsylvania, Philadelphia, PA, USA
NCT04241315	ELUCIDATE: Enabling Lung Cancer Identification Using Folate Receptor Targeting	Lung neoplasms, Lung cancer	Completed	Stamford, CT, US; University of Iowa, Iowa, USA; Beth Israel Deaconess Medical Center Boston, MA, USA (and 9 others)
NCT02629549	Intraoperative Imaging of Pituitary Adenomas by OTL	Neoplasms, Pituitary adenomas	Terminated	Hospital of the University of Pennsylvania, Philadelphia, PA, USA

**Table 2:** Other similar agents used for intraoperative tumor detection.

Agents Used for Intraoperative Molecular Imaging	Comments
5-Aminolevulinic acid	- It emits fluorescence in the visible spectrum, compared with pafolacianine, which emits fluorescence in the near-infrared spectrum [67]; - It provides an inferior depth of penetration and significantly higher background signal compared with pafolacianine [67]; - It is used predominantly for targeting bladder cancer [100] and malignant gliomas (approved by the FDA in 2017 for intraoperative molecular imaging in patients with suspected high-grade gliomas) [101]; - It requires patients to be protected from the sun and ultraviolet radiation for 24 hours after surgery, compared with pafolacianine, which does not limit patient activity or restrict discharge from the hospital [67]; - It may cause liver damage, chest pain, neuropathy, and sudden death [102], while pafolacianine shows minor side effects and no associated toxicity [6].
Folate-fluorescein isothiocyanate	- It exhibits fluorescence in the visible range, compared with pafolacianine, which emits fluorescence in the near-infrared spectrum [13]; - It overlaps with the absorption spectrum of hemoglobin, reducing the signal in a surgical field covered by blood [47]; - It binds FRα, having high specificity [60]; - It differs from pafolacianine in regard to the associated fluorochrome, the folate-fluorescein isothiocyanate carrying fluorescein, while pafolacianine carries an indocyanine green analogue called SO456; - It provides a higher background autofluorescence, inferior contrast, increased scattering, and limited tissue penetration than pafolacianine [91]; - It has significant patient safety advantages, with no associated toxicity [13].
Sodium fluorescein	- It can be visualized under visible light [103]; - It does not have a specific target, but its uptake in the cancer lesions can be estimated by the endothelial breakdown and high vascular permeability [103,104]; - It is safe, implying no toxicity [68]; - It provides a good contrast, but with an inferior depth of penetration [74,75].
Indocyanine green	- It is a near-infrared contrast agent [13]; - It is associated with a similar depth of detection and autofluorescence to pafolacianine, due to its decreased light spread and blood absorption [105,106]; - It does not have tumor specificity, and it may also accumulate in areas of inflammation, creating background autofluorescence [58]; - It maintains fluorescence for a couple of minutes, compared to pafolacianine, which exhibits fluorescence for hours [91,107]; - It exhibits few to no adverse reactions [13].

**Table 3:** Benefits and limitations.

Criteria	Benefits	Limitations
Relatively new technology	Near-infrared imaging with pafolacianine is a relatively new technology, recently approved by the FDA for tumor detection in ovarian cancer patients, which has promising outcomes in terms of safety and efficacy [6].	As a newly developed technique, it needs to be popularized among both surgeons and patients in order to reap the benefits of improved tumor detection. Hospitals also need to provide the necessary logistics to enable surgical procedures of this kind and to provide surgeons with special training on their use.
Administration	Pafolacianine is intravenously administered within a couple of hours prior to the surgery [6]. In comparison with other targeted fluorophores that are administered up to 1 week before the surgery, pafolacianine seems to be efficient even when delivered just a few hours before resection [13].	It is recommended to avoid the use of folate, folic acid, and folate-based supplements during the 48 h prior to pafolacianine administration [6].
Safety	Pafolacianine has a low toxicity profile, the most frequent side effects being dyspepsia, vomiting, nausea, abdominal pain, chest discomfort, flushing, pruritus, and hypersensitivity [6].	It can lead to fetal harm when delivered to pregnant women [6].
Efficacy	Pafolacianine binds FRα with a ∼1 nM affinity, and it is cleared from tissues which do not express the receptor, with a half-time of <30 minutes. It is demonstrated that pafolacianine enables tumor detection at concentrations of less than 100-fold those needed to cause signs of toxicity [23]. It also provides excellent contrast against the healthy background and a long residence time in the malignant lesions. It exhibits extremely low autofluorescence and a great depth of penetration, with cancer lesions being visible up to 1 cm below the tissue surface [24,108,109,110]. Additionally, intraoperative imaging using pafolacianine provided a 2-fold improvement in surgeons’ ability to identify malignant lesions [4].	Image interpretation errors may also occur, including both false negatives and false positives [6]. False positive errors can be produced due to pinolcaine’s binding of FRβ, overexpressed on the surface of the macrophages accumulated in the non-malignant regional lymph nodes [4].

## Data Availability

Not applicable.

## Abbreviations

U.S. Food and Drug Administration (FDA); magnetic resonance imaging (MRI); folate receptor alpha (FRα); folate receptor β (FR β); epithelial ovarian cancer (EOC); tumor-to-background ratio (TBR); non-small-cell lung cancer (NSCLC); ground-glass opacity (GGO); renal cell carcinoma (RCC).
